# Predictive Capacity of Boar Sperm Morphometry and Morphometric Sub-Populations on Reproductive Success after Artificial Insemination

**DOI:** 10.3390/ani11040920

**Published:** 2021-03-24

**Authors:** Vinicio Barquero, Eduardo R. S. Roldan, Carles Soler, Jesús L. Yániz, Marlen Camacho, Anthony Valverde

**Affiliations:** 1 Animal Reproduction Laboratory, School of Agronomy, Costa Rica Institute of Technology, San Carlos Campus, Alajuela 223-21002, Costa Rica; vinicio1196@gmail.com (V.B.); mcamacho@tec.ac.cr (M.C.); 2 Department of Biodiversity and Evolutionary Biology, Museo Nacional de Ciencias Naturales (CSIC), 28006 Madrid, Spain; roldane@mncn.csic.es; 3 Department of Cellular Biology, Functional Biology and Physical Anthropology, University of Valencia, Campus Burjassot, C/Dr Moliner 50, 46100 Burjassot, Spain; carles.soler@uv.es; 4 BIOFITER Research Group, Higher Polytechnic School of Huesca, Institute of Environmental Sciences of Aragón (IUCA), University of Zaragoza, Ctra. Cuarte s/n, 22071 Huesca, Spain; jyaniz@unizar.es

**Keywords:** sperm, CASA-morph, fertility, sperm subpopulations, boar, sow, litter size

## Abstract

**Simple Summary:**

The efficiency of swine production measured as litter size influences the profitability of the pig industry. Furthermore, sow fertility potential depends in part on the boar semen quality and reproductive efficiency. The objective of this study is to compare boar sperm head size and morphometric features of shape to evaluate their relationships with reproductive success after artificial insemination (AI). A morphometric analysis of boar ejaculate reveals morphometrically separate sub-populations. The differences between sub-populations are displayed for sperm head size. In addition, sperm clustering into sub-populations did not have a predictive capacity on litter size variables. Nevertheless, the morphometric variables of the sperm may have a predictive, albeit reduced, capacity regarding litter size variables. The results of this study therefore open up possibilities for future assessments of fertility.

**Abstract:**

The aim of the study was to compare the morphometric features of sperm head size and shape from the Pietrain line and the Duroc × Pietrain boar crossbred terminal lines, and to evaluate their relationship with reproductive success after artificial insemination of sows produced from crossbreeding the York, Landrace and Pietrain breeds. Semen samples were collected from 11 sexually mature boars. Only ejaculates with greater than 70% motility rate and <15% of abnormal sperm were used for artificial inseminations (AI) and included in the study. Samples were analyzed using an ISAS^®^v1 computer-assisted sperm analysis system for eight morphometric parameters of head shape and size (CASA-Morph). Sub-populations of morphometric ejaculates were characterized using multivariate procedures, such as principal component (PC) analysis and clustering methods (k-means model). Four different ejaculate sub-populations were identified from two PCs that involved the head shape and size of the spermatozoa. The discriminant ability of the different morphometric sperm variables to predict sow litter size was analyzed using a receiver operating characteristics (ROC) curve analysis. Sperm head length, ellipticity, elongation, and regularity showed significant predictive capacity on litter size (0.59, 0.59, 0.60, and 0.56 area under curve (AUC), respectively). The morphometric sperm sub-populations were not related to sow litter size.

## 1. Introduction

The efficiency of pig livestock production, measured as piglets born alive, total born per litter, and the number of piglets weaned by a sow per year, influence the profitability of the pig industry [1]. Sow fertility potential depends in part on the semen quality of the boar [2]. The ability to identify sub-fertile boars to improve reproductive efficiency has its basis in economic profitability [3]. The classical routine evaluation of boar fertility has traditionally been based on the assessment of semen variables, including seminal volume, sperm concentration, motility, and morphology [3]. Boars of different breeds, lines, or crossbreeds can produce ejaculates of different volumes, sperm concentrations, motilities, and kinematic patterns [4,5]. Sperm morphometry differs according to the breed of the male [6]. It has been reported that considerable variability exists in sperm morphometry among males from the same population [7,8,9,10]. Furthermore, the sperm morphometry variability of different ejaculates from the same animal has been documented [11]. Interest in the size and shape morphometry of sperm heads has led to intense research in recent years [12,13]. The morphometric variables of the size and shape of the sperm head exhibit a relevant variability among species, among males of different species [14,15], and between breeds or selected lines within a species [6,10]. Some studies have described a correlation between the fertility of males used for artificial insemination (AI) and sperm morphometry [12], and head size has been associated with factors predisposing fertility [16]. Other authors refer to an association between the head size and shape of the spermatozoa and semen or ejaculate variables [17,18,19]. The total spermatozoa in the ejaculate have been associated with the morphometric variables of spermatozoa: ejaculates with a low sperm concentration have smaller, shorter, and narrower head sizes and a smaller head area than those of ejaculates with a high sperm count [8]. Current computer-assisted sperm morphometric analysis CASA-Morph systems can be used to analyze individual sperm morphometrics more accurately, and this information can be submitted to a multivariate procedure such as cluster analysis for an overview of distinct sperm patterns grouped into sub-populations (SPs) or clusters [20]. Some authors have indicated that the distribution of spermatozoa in each sperm sub-population can vary among males, and some of these sub-populations have been correlated with sperm quality [21]. Several studies have examined the association between sperm head size and fertility in pigs [6,16,22] and species such as cattle [23], buffalo [24], sheep [25], goats [26], horses [27], dogs [28]. or humans [29]. A limited number of studies have focused on the size and head shape of sperm and the characterization of sperm sub-populations on litter size in livestock species such as pigs [30,31]. Even today, the biological relevance and direct biological meaning of sperm sub-populations remain controversial. The aim of the present study was to compare the morphometric features of the head size and shape of spermatozoa from the Pietrain and Duroc × Pietrain boar crossbred terminal lines, and to evaluate their relationships with the litter size of sows produced from crossbreeding of the York, Landrace, and Pietrain breeds.

## 2. Materials and Methods

### 2.1. Animals 

The experiment was conducted at a commercial swine farm (Agropecuaria Los Sagitarios S.A., Alajuela, Costa Rica) during 2019–2020 in the northwest of Costa Rica (Río Cuarto, 10°20′32″ N, 84°12′55″ W, Alajuela, Costa Rica, Central America) following the laws and regulations controlling experiments on live animals in Costa Rica. This study was performed following ethical principles, and with the approval of the Committee of Centro de Investigación y Desarrollo de la Agricultura Sostenible para el Trópico Húmedo at the Costa Rica Institute of Technology (CIDASTH-ITCR) according to Section 08/2020, article 1.0, DAGSC-100-2020. Eleven sexually mature and healthy boars from two commercial terminal male lines (ML: Duroc × Pietrain (*n* = 8) and Pietrain boars (*n* = 3)), 20.9 ± 3.0 (Pietrain) and 24.1 ± 9.8 (Duroc × Pietrain) months of age at the beginning of the experiment and of known fertility were used as semen donors in this study. For the study, the breeding boars were housed individually in well-ventilated pens with an average temperature of 25.6 ± 2.94 °C during the time of the experiment. The females came from four crossbred genetic lines (FL: York (Y), Landrace (L) and Pietrain (P), with the crossing schemes YLP-50 (¼ Y × ¼ L × ½ P), YLP-75 (^1^/_8_ Y × ^1^/_8_ L × ^3^/_4_ P), YLP-87.5 (^1^/_16_ Y × ^1^/_16_ L × ^7^/_8_ P), and Y-L-50 (½ Y × ½ L)). All of the females were bred on the farm and they came from maternal crossing schemes. Animals were fed with a standard breeder mixture (made on the farm) containing maize, soybean meal, mineral mixture, and common salt, as ingredients to fulfill the nutrient requirements according to the Nutrient Requirements of Swine [32]. Pregnant sows were provided with 2.5 kg of concentrated feedstuff in the first 2/3 of the gestation period and 3 kg in the final third. Males consumed 2.5 kg of concentrated feedstuff per day and were provided with water ad libitum. The gilts inseminated were 7 months of age, with a minimum weight of 145 kg.

### 2.2. Fertility Trial

A total of 816 triple-artificial inseminations, performed with homospermic ejaculates, were evaluated on 272 sows. These AIs were conducted aleatory with 40 ejaculates from eleven males. That means that each genetic line of females was randomly inseminated with each genetic line of males. Each ejaculate was used in the first 3 days post-collection, and only those used to inseminate at least three females were evaluated. The mean of sows inseminated per boar was 24.7 ± 10.1 females. These AI resulted in 3.99 ± 3.16 farrowings from each female. Parameters measured at the time of farrowing were: litter size as total piglets born (TPB) per litter, piglets born alive (PBA), piglets born dead (PBD), number of mummies (MP), and litter weight (LW; kg).

### 2.3. Collection and Examination of Semen

Semen samples were collected in the morning, once per week, using the “gloved-hand” technique [33], and immediately placed into a water bath at 37 °C at the farm laboratory. In all cases, the sperm-rich fractions were collected and diluted with a commercial extender (Zoosperm ND5; Import-Vet, Barcelona, Spain). Insemination doses contained 3.7 ± 1.3 × 10^9^ spermatozoa. From each boar, 3.64 ± 0.81 ejaculates were obtained. Samples from each ejaculate were evaluated for motility, and only ejaculates with at least 70% motile spermatozoa and 85% morphologically normal spermatozoa were used. The concentration was measured with Spermacue (Minitube, GmbH, Tiefenbach, Germany), following established protocols. Samples were stored at 17 °C and then transported to the laboratory in the same refrigerated conditions used for commercial distribution (17 °C). A volume of one milliliter (1 mL) of mixed sample was placed in an Eppendorf^®^ tube (Sigma-Aldrich, St. Louis, MO, USA) and maintained at 37 °C for 30 min before use.

### 2.4. Sample Preparation for Morphometric Analysis

Duplicate samples for morphometric analysis were prepared from the ejaculates of each commercial line. After being mixed, 10 μL of each sample was spread on a glass slide and subsequently air-dried. The slides were stained using the Diff-Quik^®^ kit (Medion Diagnostics, Düdingen, Switzerland), following the instructions of the manufacturer. All the slides were identified and then analyzed in a double-blind scheme.

### 2.5. Assessment of Sperm Variables

Microscope slides were analyzed for sperm head morphometry using the ISAS^®^ v1 (Integrated Semen Analysis System, Proiser R+D, Valencia, Spain). The equipment comprised of a UB203 microscope (UOP/Proiser R+D) equipped with a bright-field 100× objective and a 3.3× photo-ocular. A digital video camera (Proiser 782 m, Proiser R+D) was mounted on the microscope to capture the images and transmit them to the computer. The array size of the video frame grabber was 746 × 578 × 8 bit, providing a resolution of the analyzed images of 0.084 μm/pixel in both axes, and 256 gray levels. The resolution of the images was 0.08 μm per pixel in both the horizontal and vertical axes. The sperm heads were captured randomly in different fields, and only those that overlapped with background particles or other cells so as to interfere with the subsequent image processing were rejected. An Initial erroneous definition of the sperm head boundary was corrected by varying the analysis factor of the system. When it was not possible to obtain a correct boundary, the sperm head was deleted from the analysis.

For the analysis of motility, ISAS^®^ D4C20 disposable counting chambers (Proiser R+D., Paterna Spain) were used after being pre-warmed to 37 °C. After homogenization of the samples, a volume of 3 μL was distributed along the counting chamber race by capillarity to fill it completely. Analyses were conducted using the CASA-Mot system of an ISAS^®^ v1 (Proiser R+D S.L., Paterna, Spain). The frame rate used was 50 Hz, with the final resolution of the images being 746 × 578 pixels. The camera was attached to a UB203 microscope (UOP/Proiser R+D) with a 1× eyepiece and a 10× negative phase contrast objective (AN 0.25) and an integrated heated stage was maintained at a constant temperature of 37 ± 0.5 °C. The CASA settings used were: a particle area between 10 and 80 μm^2^, and connectivity of 11 μm. The percentage of total motile cells and progressive motility (%) corresponded to the spermatozoa swimming forward quickly in a straight line. The parameters defining progressive motility were straightness (STR) ≥ 45%, and average path velocity (VAP) ≥ 25 μm/s, defined as the average velocity over a smoothed cell path.

A single technician carried out the assessments of sperm morphology. Sperm were classified as having normal or abnormal morphologic features following WHO strict criteria [34]. A total of 200 sperm were analyzed per slide, and 100 sperm from each of two different locations on the slide were assessed. If the difference between the percentage of normal sperm in the two areas was 5 percentage points or lower, the mean value was calculated.

### 2.6. Morphometric Analysis

Images from about 200 spermatozoa from each sample were captured and analyzed to obtain eight morphometric variable values. Following the criteria of Boersma [35], the sperm heads were measured on each slide for four primary parameters of head size, length (L, μm), width (W, μm), area (A, μm^2^), and perimeter (P, μm); and four derived dimensionless parameters of head shape, ellipticity (L/W), rugosity (4πA/P^2^), elongation ((L − W)/(L + W)), and regularity (πLW/4A). Data from each individual sperm cell were saved in a database compatible with Excel^®^ (Microsoft Corporation, Redmond, Washington, DC, USA) by the software for further analysis.

### 2.7. Statistical Analysis

The data obtained for the analysis of all sperm parameters were first assessed for normality and homoscedasticity using the Shapiro–Wilks and Levene tests. A normal probability plot was used to assess normal distribution. Multivariate analyses were performed to identify sperm sub-populations from the set of ejaculate sperm morphometric data. All of the values for the morphometric variables were standardized to avoid any scale effect or variables with larger scales from dominating how clusters were defined. Thus, all variables were considered by the algorithm to be of equal importance. The standardization used was Z-score, and it transformed data by subtracting the mean value for each field from the values of the file, and then dividing by the standard deviation of the field, resulting in data with a mean of zero and a standard deviation of one. Differences between means were analyzed using a Bonferroni test. Results are presented as mean ± standard deviation (s.d.). Statistical significance was considered at *p* < 0.05. All data were analyzed using the IBM SPSS software package version 23.0 for Windows (SPSS Inc., Chicago, IL, USA).

#### 2.7.1. Multivariate Procedures

A subset of the data was created using the means per ejaculate of all eight morphometric variables. The first process carried out was a principal component analysis (PCA) of the morphometric data to derive a small number of linear combinations that still retained as much information as possible from the original variables. The number of principal components (PC) used in the next part of the analysis was determined using the Kaiser criterion, namely selecting only those with an eigenvalue (variance extracted of each PC) > 1. Furthermore, Bartlett’s sphericity test and the Kaiser–Meyer–Olkin (KMO) measure were performed [36]. As a rotation method, the varimax method with Kaiser normalization was used [37].

An analysis was conducted to classify the ejaculates into a reduced number of subpopulations (clusters), based on the scores obtained from the factor analysis. This was accomplished in two phases combining hierarchical and non-hierarchical clustering procedures. The process to perform a non-hierarchical analysis was conducted with a k-means model that used Euclidean distances from the quantitative variables after the standardization of these data, so the cluster centers were the means of the observations assigned to each cluster [38]. The multivariate k-mean cluster analysis was made to classify the ejaculates into a reduced number of sub-populations (clusters) according to their morphometric variables. In the final process, to determine the optimal number of clusters, the final centroids were clustered hierarchically using the Ward method [39]. Thus, the clustering procedure enabled the identification of sperm sub-populations because each cluster contributed to a final cluster formed by the ejaculate linked to the centroids. Then, ANOVA procedures were applied to evaluate statistical differences in the distributions of observations (individual spermatozoa) within the sub-populations, and then a generalized linear model (GLM) procedure was used to determine the effects of the genetic lines of the boar and sow breeds on the mean morphometric variable values defining the different sperm sub-populations (e.g., the cluster centers).

#### 2.7.2. ROC Analysis

The diagnostic test with a dichotomous outcome (positive/negative fertility test results) of the different morphometric semen variables to predict litter size variables was analyzed using receiver operating characteristic (ROC) curve analysis. This diagnostic test evaluation used sensitivity and specificity as measures of test accuracy when compared with a standard status (farrowing). The sensitivity (true positive rate) and specificity (true negative rate) of each morphometric parameter varied across the different thresholds, and the sensitivity was inversely related to the specificity. The plot of sensitivity versus the 1-specifity is called the receiver operating characteristic (ROC) curve and the area under the curve (AUC). AUC varies from 0.5 (test with no discriminatory ability) to 1 (perfect discriminatory ability). A ROC was also used to calculate the elective breaking point (cut-off value) of each morphometric sperm variable. The analysis may also be used to determine the optimal cut-off value (optimal decision threshold).

## 3. Results

### 3.1. Overall Semen Variables

The sperm concentration, the volume of semen, and the total spermatozoa in the ejaculate were 374.23 ± 129.24 × 10^6^/mL, 231.98 ± 63.08 mL, and 82.04 ± 23.73 × 10^9^, respectively. The sperm concentration (million/mL) was 378.63 ± 134.98 in the Duroc × Pietrain crossbreed and 361.00 ± 112.35 in the Pietrain. The total motility (%) of the boar samples was 77.36 ± 11.17, with an overall range of 35.05–93.69%. The progressive motility of the sperm (%) was 63.76 ± 11.96. The average total motility (%) for the Duroc × Pietrain and the Pietrain boars was 81.28 ± 7.76, and 65.61 ± 11.73, respectively. The progressive motility (%) was 67.00 ± 10.05 (Duroc × Pietrain), and 54.04 ± 12.19 (Pietrain).

### 3.2. Morphometric Variables

**Figure 1:**
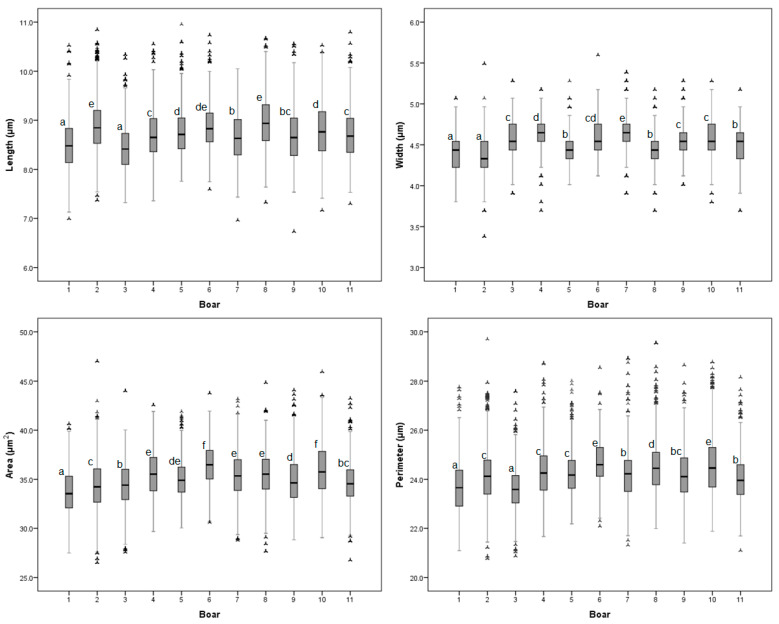
Box and whisker plot of distribution of boar sperm head size morphometric variables. Each box contains the central 50% of the observations and the whisker contains the central 95%. ^a–f^ Boxes labelled with different letters indicate differences between boars. *p* < 0.05.

There was an animal effect on the sperm head size variables (*p* < 0.05; Figure 1; supplementary Table S1). The sperm head size from the Duroc × Pietrain boars was, in fact, larger than the sperm head size of the Pietrain boars. The sperm from the Duroc × Pietrain boar ejaculates had a larger head perimeter (0.5 μm) and head area (1.26 μm^2^) than the sperm from the Pietrain boar ejaculates (*p* < 0.05). The Duroc × Pietrain ejaculates contained longer (0.15 μm) and wider (0.08 μm) sperm than did the Pietrain ejaculates (*p* < 0.05). There were no differences in the shape of the sperm heads between the boar breeds and the AI semen doses used for females insemination (Table 1).

The indices used to evaluate the differences in the dimension of sperm from ejaculates indicate differences between the males in head size. The percentage of variation of the morphometric traits between the ejaculates with the lowest and highest sperm head sizes were 5.02% for length, 6.17% for width, 5.31% for the area, 4.09% for perimeter, 9.76% for ellipticity, and 3.90% for rugosity. The sperm doses used on the YLP-75 sows evaluated had a larger sperm head size (*p* < 0.05) than the sperm doses on the YLP-50 sows (0.44 μm^2^ larger on average). There were no differences (*p* > 0.05) for the sperm head size variables between the YLP-87.5 and Y-L-50 female lines (Table 1).

### 3.3. Fertility Traits

**Figure 2:**
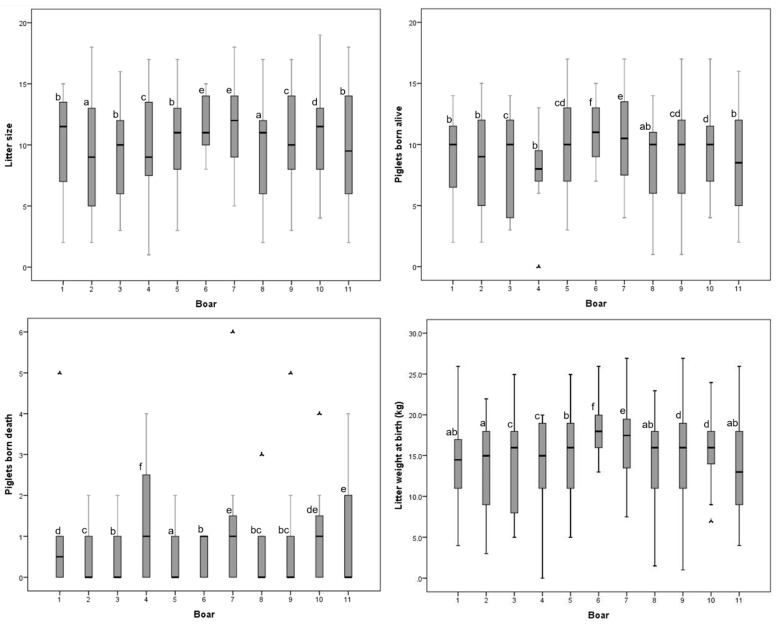
Box plot of distribution of litter size and other litter variables for the different boars. Each box contains the central 50% of the observations, and the whisker contains the central 95%. ^a–f^ Boxes labelled with different letters indicate differences between boars. *p* < 0.05.

The mean fertility rate was 69.6 ± 21.67%. There were no differences between the male lines for this variable. The Sows inseminated with the Duroc × Pietrain semen had a larger (*p* < 0.05) total number born per litter (10.53 ± 3.94) than those inseminated with the Pietrain semen (9.72 ± 4.24). In the Duroc × Pietrain crossbreed, the number of piglets born alive (PBA = 9.46 ± 3.70) and piglets born dead (PBD = 0.80 ± 1.11) were higher than those of the Pietrain boars (PBA = 8.89 ± 3.77, PBD = 0.65 ± 0.95; *p* < 0.05). Litter weight at birth and number of mummies were lower for the Pietrain boars (*p* < 0.05; Table 2). There was an animal effect on litter size variables and litter weight at birth (*p* < 0.05; Figure 2; supplementary Table S2).

Fertility traits such as total born per litter (10.76 ± 4.55), piglets born alive (9.64 ± 4.30), and the number of mummies (0.35 ± 0.75; *p* < 0.05) were higher in the Y-L-50 crossbred females than in the other lines. The YLP-87.5 crossbred sows presented a lower (*p* < 0.05) number of piglets born dead (0.55 ± 1.00). The YLP-50 sows had fewer piglets born alive (*p* < 0.05). The litter weight at birth was higher in the YLP-75 sows (Table 2).

### 3.4. Sub-Population Structure

**Figure 3:**
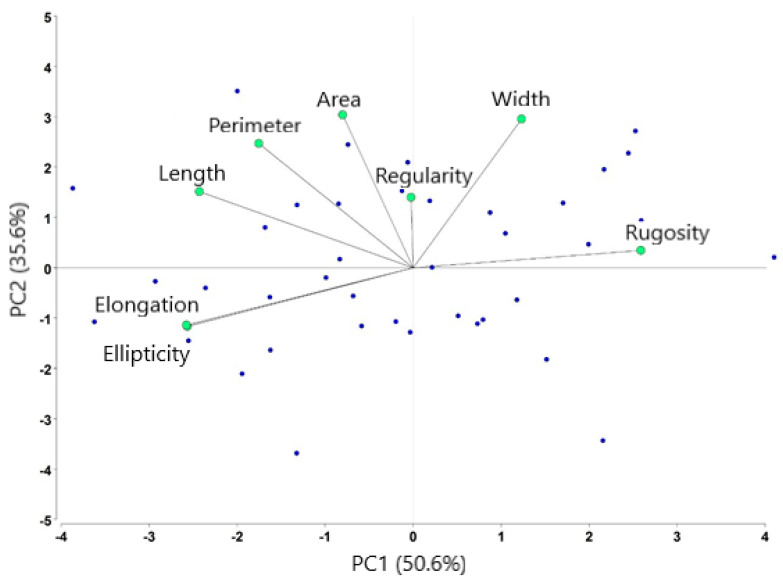
Distribution of morphometric variables after principal component analysis. Each blue point represents an ejaculate. Four sperm sub-population were identified from forty ejaculates.

Results from the principal component analysis revealed two PC factors. PC1 was referred to as “head shape,” or long, stretched, tubiform cells, which were represented by the elongation, ellipticity, head length, and rugosity (in reverse order). The larger eigenvector corresponded to elongation (0.47). PC2 was represented by the sperm head area, perimeter, and width, and was named “head size.” It is mainly related to the head area (Eigenfactor = 0.55) (Table 3). These results indicate that the shape and head size of sperm have a relatively greater effect (86.2%) on the total variance explained, and the elongation and ellipticity showed a maximum correlation (Figure 3).

Four sperm subpopulations (SPs) with different morphometric patterns were obtained from the morphometry traits. Summary data for the morphometrics and SP fertility are presented in Table 4. They can be summarized as follows: sub-population 1 (SP1) included ejaculates with lower values for ellipticity (1.83 ± 0.03) and the widest heads (4.69 ± 0.06). This population represented 17.5% of the total sperm from ejaculates. Sub-population 2 (SP2) contained the highest values for head area and perimeter, with values of 35.77 ± 1.24, and 24.48 ± 0.44, respectively. About 42.5% of the spermatozoa from ejaculates were assigned to this sub-population. Sub-population 3 (SP3) included 20.0% of the sperm from all ejaculates, and was represented by shorter length (8.49 ± 0.22), with a smaller area (34.16 ± 1.07) and head perimeter (23.72 ± 0.51) values. This population had intermediate head shape values indicated by the ellipticity, elongation, and regularity values. Sub-population 4 (SP4) contained 20.0% of the spermatozoa from the total ejaculate population, and these spermatozoa had the highest values for head length (8.88 ± 0.13), ellipticity (2.03 ± 0.02), and elongation (0.34 ± 0.01) (Table 4).

### 3.5. Predictive Capacity of Fertility

The morphometric variables of the sperm with significant results in the ROC curve analysis are presented in Table 5. Sperm head length, ellipticity, elongation, and regularity showed significant, albeit limited, predictive capacity on the litter size variables (range: 0.56–0.60 AUC). Similarly, the sperm subpopulations showed limited predictive capacity on the litter size variables (data not shown). Cut-off points, with their sensitivities and specificities, are also presented in Table 5. Among the sperm shape variables, the best cut-off points to identify ejaculates with low fertility potential in relation to the number of mummies were 8.71 μm for head length, 1.92 for ellipticity, and 0.31 for elongation.

## 4. Discussion

Fertility is a complex trait in which a wide range of sperm characteristics may be involved [40]. These fertility parameters can be expressed by several variables with distributions that can be continuous—where any value in the range of the distribution is possible (farrowing rate), or discrete—where only specific values can be returned (piglets born alive). The results of the present study indicate that the Pietrain x Duroc crossbreed had larger sperm heads than the Pietrain boars. Similar results have been described in the larger spermatozoa head length, area, and perimeter of crossbred boars, as compared with purebred boars [41]. The morphometric variables related to the head size of the sperm were also significantly different among the individual Duroc × Pietrain or Pietrain boars, and between the Duroc × Pietrain and Pietrain boars. In stallions, differences in sperm head size within breeds [42,43] and between stallions [44] have been reported. Moreover, our results also showed that the sperm head size increased as the sperm concentration of the ejaculate increased, in agreement with previous studies in pigs [45]. In other species, such as dogs, it has been found that sperm concentration can influence the head size dimensions [46]. The head size and shape of the sperm may affect their motility [47] and fertilization capacity [45]. Overall, the percentage of motile sperm presented by the Pietrain males was lower than that of the Duroc × Pietrain boars. 

Sperm length has been positively correlated with motility [48], and an inverse relationship has been described between sperm length and the effective time of maintenance of sperm cell motility, and thus its capacity for oocyte fertilization [49]. In pigs, lower-fertility boars also showed more elongated sperm heads [22]. Our study reports that boars with a larger litter size had significantly less elongated spermatozoa. Nevertheless, the mortality of piglets was greater in these males. This observation is in agreement with a previous study of Pietrain boars, in which the sperm heads of high-fertility boars were less elongated and smaller than those of lower fertility boars [22]. Although the sperm head morphometric parameters explained a variation in the litter size of 7.7%, these results are partially explained by factors affecting the female [50]. The head shape also can affect the hydrodynamics of the spermatozoa, as sperm with elongated heads can move faster than those with more elliptical heads [51], and elongated heads have a relationship with male fertility rates [52]. Some authors have suggested that sperm with higher ellipticity values (head length/width) presented a lower progressiveness [53]. Our results indicate that there was a greater progressiveness in the Duroc × Pietrain boars than in the Pietrain boars, but no differences were found between the ellipticity of these two male lines. In humans, it has been observed that morphologically normal spermatozoa showed a faster acrosome reaction than the tapered, large, and small-headed types of spermatozoa [54,55]. Thus, morphological structure and functionality have a close relationship [12,55].

The outcome of this work indicated that crossbreeding influenced the head size and shape of boar sperm cells. The sperm from the Pietrain-line ejaculates had shorter and narrower heads, with a smaller head area and perimeter than sperm from the Duroc × Pietrain line. There are genetic factors to modeling shape and sperm head size [6,7,41]. The present study shows differences between the genetic lines of males for sperm head size. In rams, the distribution of sperm sub-populations had been associated with intra- and inter-male differences [56]. While determining that morphometric differences occur between two male lines is biologically notable, it can be difficult to clearly connect these morphometric measurements to the boar semen used in assessments of sow fertility when artificially inseminated. In our study, this was most evident when the head shape morphometry was reviewed. Studies in humans demonstrated that the size and normal shape of sperm heads affect functions, such as the acrosome reaction [57] and the binding zone to the zona pellucida of the oocyte [58], which could affect the potential fertility of the male [59]. 

Sperm head size has also been related to fertility. Accordingly, variability in sperm head size has been correlated with variation in the chromatin structure of the cell nucleus [23]. Other authors have suggested that minor variations in the shape of sperm heads can be associated with changes in the chromatin structure in the spermatozoa nucleus, which can result in reduced fertility [60]. When the multivariate analysis was applied to the ejaculates analyzed for fertility variables and the spermatozoa size and head shape, discrete sub-populations (cluster centers) were generated based on the set number of two standardized principal components. In the analysis of sub-populations of sperm head dimensions, significant differences in the values for length, width, area, and perimeter were found between the sub-populations of Duroc × Pietrain and Pietrain boar sperm heads. The differences in the cluster populations were analyzed across all boar semen morphometric variables for size, head shape, and semen doses used in AI. Several studies have described the presence of different sub-populations within an ejaculate [11,19,61,62,63,64,65,66,67]. These sub-populations may be affected by external factors associated with semen, such as extender type or species [68,69]. Even the sperm sub-population distribution can vary depending on the statistical multivariate procedure used [70]. In boars, different morphometric sperm sub-populations were found and were categorized according to head size as large, small elongated, and small round, and these variables could have a functional involvement [31]. Past studies have associated sperm head morphometry and fertility variables in boars [22], male goats [26,71], stallions [27], rams [52], and rabbits [72], in which subsequent fertility was reduced with the lowest head size variables. When comparing male lines, we found that that boars with lower sperm head sizes generally had higher litter sizes. These findings were verified when we analyzed the sperm sub-populations in their ejaculate. The sperm sub-populations SP2 and SP3 presented more minor variations amongst each other than the sub-populations SP1 and SP4 did. Thus, there was less uniformity regarding the sperm head size variables between SP1–SP4, which suggests the idea that possibly the sperm competition between these sub-populations supports low levels of sperm competition, which could result in poor semen quality, as has been described in several studies on rodents [73,74].

## 5. Conclusions

We have shown that morphometric analysis of boar ejaculates reveals morphometrically separate populations. Differences between sub-population sperm head sizes were displayed. Sperm morphometric variables may have a predictive capacity on the litter size variables. Clustering the sperm into sub-populations did not have a predictive capacity on the litter size variables.

## Figures and Tables

**Table 1:** Morphometric variables (mean ± s.d.) of boar sperm size and head shape of in different crossbred males and AI doses use in sows.

	Boar		Sows			
Variable	Pietrain	D × P	YLP-50	YLP-75	YLP-87.5	Y-L-50
Length	8.62 ± 0.60 ^α^	8.77 ± 0.53 ^β^	8.70 ± 0.52 ^a^	8.78 ± 0.55 ^c^	8.74 ± 0.53 ^b^	8.75 ± 0.56 ^b^
Width	4.46 ± 0.23 ^α^	4.54 ± 0.22 ^β^	4.50 ± 0.23 ^a^	4.53 ± 0.22 ^c^	4.51 ± 0.23 ^b^	4.51 ± 0.22 ^b^
Area	34.20 ± 2.47 ^α^	35.46 ± 2.39 ^β^	34.94 ± 2.33 ^a^	35.38 ± 2.53 ^c^	35.08 ± 2.46 ^b^	35.07 ± 2.28 ^b^
Perimeter	23.84 ± 1.13 ^α^	24.34 ± 1.06 ^β^	24.15 ± 1.03 ^a^	24.31 ± 1.11 ^c^	24.23 ± 1.05 ^b^	24.19 ± 1.06 ^ab^
Ellipticity	1.94 ± 0.17	1.94 ± 0.14	1.94 ± 0.15	1.94 ± 0.14	1.95 ± 0.15	1.94 ± 0.15
Rugosity	0.76 ± 0.04	0.75 ± 0.04	0.75 ± 0.04	0.75 ± 0.03	0.75 ± 0.04	0.75 ± 0.04
Elongation	0.32 ± 0.04	0.32 ± 0.03	0.32 ± 0.02	0.32 ± 0.03	0.32 ± 0.03	0.32 ± 0.04
Regularity	0.88 ± 0.03	0.88 ± 0.04	0.88 ± 0.03	0.88 ± 0.04	0.88 ± 0.04	0.88 ± 0.03

AI: artificial insemination, s.d.: standard deviation, length (L, μm), width (W, μm), area (A, μm^2^), perimeter (P, μm), ellipticity (L/W), rugosity (4πA/P^2^), elongation ((L − W)/(L + W)), regularity (πLW/4 A). Y: York, L: Landrace, P: Pietrain, D: Duroc. YLP-50 = (¼ Y × ¼ L × ½ P), YLP-75 = (^1^/_8_ Y × ^1^/_8_ L × ^3^/_4_ P), YLP-87.5 = (^1^/_16_ Y × ^1^/_16_ L × ^7^/_8_ P), Y-L-50: ^1^/_2_ Y × ^1^/_2_ L. ^α,β^ Different letters indicate differences between crossbred males. ^a–c^ Different letters indicate differences between crossbred females. *p* < 0.05.

**Table 2:** Fertility variables (mean ± s.d.) of litter size and piglet mortality of according to source of boar semen and in different lines of sows.

	Boar		Sows			
Variable	Pietrain	D × P	YLP-50	YLP-75	YLP-87.5	Y-L-50
Total born per litter	9.72 ± 4.24 ^α^	10.53 ± 3.94 ^β^	10.12 ± 3.54 ^a^	10.37 ± 4.03 ^b^	10.01 ± 3.89 ^a^	10.76 ± 4.55 ^c^
Piglets born alive	8.89 ± 3.77 ^α^	9.46 ± 3.70 ^β^	8.71 ± 3.39 ^a^	9.26 ± 3.53 ^b^	9.25 ± 3.64 ^b^	9.64 ± 4.30 ^c^
Piglets born dead	0.65 ± 0.95 ^α^	0.80 ± 1.11 ^β^	1.13 ± 1.08 ^d^	0.88 ± 1.33 ^c^	0.55 ± 1.00 ^a^	0.77 ± 0.69 ^b^
Number of mummies	0.19 ± 0.35 ^α^	0.26 ± 0.64 ^β^	0.28 ± 0.6 ^b^	0.23 ± 0.56 ^a^	0.22 ± 0.51 ^a^	0.35 ± 0.75 ^c^
Litter weight at birth	14.23 ± 5.74 ^α^	15.58 ± 5.59 ^β^	13.95 ± 4.75 ^a^	15.44 ± 5.43 ^c^	15.16 ± 5.72 ^b^	15.05 ± 6.27 ^b^

s.d.: standard deviation. Y: York, L: Landrace, P: Pietrain, D: Duroc. YLP-50 = (¼ Y × ¼ L × ½ P), YLP-75 = (^1^/_8_ Y × ^1^/_8_ L × ^3^/_4_ P), YLP-87.5 = (^1^/_16_ Y × ^1^/_16_ L × ^7^/_8_ P), Y-L-50: ^1^/_2_ Y × ^1^/_2_ L. Litter weight at birth (kg). ^α,β^ Different letters indicate differences between crossbred males. ^a–d^ Different letters indicate differences between crossbred females. *p* < 0.05.

**Table 3:** Eigenvectors of principal components (PCs) * for the morphometric variables of boar sperm size and head shape.

Variable	PC_1_	PC_2_
Length	0.44	
Width		0.54
Area		0.55
Perimeter		0.45
Ellipticity	0.46	
Rugosity	−0.46	
Elongation	0.47	
Regularity		
Var Exp	50.6	35.6

Var Exp: variance explained in each PC. Total variance explained = 86.2%. * Expresses the more important variables in each PC. Only eigenvectors > 0.4 are presented.

**Table 4:** Morphometry of sperm heads, head shape, and fertility variables (mean ± s.d.) of the four ejaculate sub-populations (SPs) defined from boar semen samples.

Variable	SP_1_	SP_2_	SP_3_	SP_4_
Proportion of all ejaculates (%)	17.50	42.50	20.00	20.00
Length	8.57 ± 0.13 ^b^	8.81 ± 0.18 ^a^	8.49 ± 0.22 ^b^	8.88 ± 0.13 ^a^
Width	4.69 ± 0.06 ^a^	4.58 ± 0.07 ^b^	4.43 ± 0.04 ^c^	4.36 ± 0.07 ^d^
Area	35.63 ± 0.69 ^a^	35.77 ± 1.24 ^a^	34.16 ± 1.07 ^b^	34.65 ± 0.92 ^b^
Perimeter	24.04 ± 0.27 ^b^	24.48 ± 0.44 ^a^	23.72 ± 0.51 ^b^	24.20 ± 0.32 ^b^
Ellipticity	1.83 ± 0.03 ^c^	1.93 ± 0.04 ^b^	1.91 ± 0.05 ^b^	2.03 ± 0.02 ^a^
Rugosity	0.78 ± 0.01 ^a^	0.76 ± 0.01 ^b^	0.76 ± 0.01 ^b^	0.75 ± 0.01 ^c^
Elongation	0.29 ± 0.01 ^c^	0.32 ± 0.01 ^b^	0.31 ± 0.01 ^b^	0.34 ± 0.01 ^a^
Regularity	0.88 ± 0.01 ^a^	0.88 ± 0.01 ^a^	0.87 ± 0.00 ^b^	0.88 ± 0.01 ^a^

s.d.: standard deviation. Number of ejaculates = 40. Length (L, μm), width (W, μm), area (A, μm^2^), perimeter (P, μm), ellipticity (L/W), rugosity (4πA/P^2^), elongation ((L − W)/(L + W)), regularity (πLW/4A). ^a–d^ Different letters indicate differences between ejaculate sub-populations for morphometric variables. *p* < 0.05.

**Table 5:** Cut-off values of morphometric sperm variables significantly related to litter size variables calculated from receiver operating characteristic (ROC) curves.

Variable	Cut-Off Value	Sensitivity (%)	Specificity (%)	Area ROC	*p*-Value
Total born per litter					
Area	35.18	67.19	27.08	0.54	0.11
Perimeter	24.26	68.66	28.26	0.55	0.10
Piglets born alive					
Area	35.18	67.19	27.08	0.53	0.17
Perimeter	24.26	68.66	28.26	0.54	0.13
Ellipticity	1.94	67.55	26.45	0.54	0.13
Piglets born dead					
Regularity	0.88	73.02	32.19	0.56	0.04
Number of mummies					
Length	8.71	71.74	31.34	0.59	0.02
Ellipticity	1.92	73.08	33.62	0.59	0.02
Elongation	0.31	73.08	33.62	0.60	0.02

Length (L, μm), width (W, μm), area (A, μm^2^), perimeter (P, μm), ellipticity (L/W), rugosity (4πA/P^2^), elongation ((L − W)/(L + W)), regularity (πLW/4A).

## Data Availability

The data presented in this study are available within the article and/or its Supplementary Materials.

## Supplementary Materials

The following are available online at https://www.mdpi.com/2076-2615/11/4/920/s1, Table S1: morphometric variables (mean ± SEM) of the sperm size and sperm head shape of individual boars. Table S2: fertility variables (mean ± SEM) of litter size and mortality of piglets born after artificial insemination with the semen of different boars.
