# Supplementary material for: Predictive Capacity of Boar Sperm Morphometry and Morphometric Sub-Populations on Reproductive Success after Artificial Insemination
Source: Animals (Basel). 2021 Mar 24;11(4):920. doi: 10.3390/ani11040920 (PMC8064074; doi:10.3390/ani11040920)
Supplement: Supplementary file 1 [file animals-11-00920-s001.pdf]

SUPPLEMENTARY MATERIALS

Table S1. Morphometrics variables (mean ± SEM) of sperm size and sperm head shape of individual boars.

	Length	Width	Area	Perimeter	Ellipticity	Rugosity	Elongation	Regularity
1	8.51±0.02[a]	4.41±0.01[a]	33.73±0.09[a]	23.70±0.04[a]	1.93±0.01[cd]	0.76±0.01[cd]	0.32±0.01[cd]	0.87±0.01[a]
2	8.96±0.02[e]	4.38±0.01[a]	34.80±0.05[c]	24.34±0.03[c]	2.05±0.01[g]	0.74±0.01[a]	0.34±0.01[g]	0.89±0.01[cd]
3	8.45±0.02[a]	4.58±0.01[c]	34.41±0.07[b]	23.60±0.04[a]	1.85±0.01[a]	0.78±0.01[f]	0.30±0.01[a]	0.88±0.01[bc]
4	8.71±0.02[c]	4.62±0.01[d]	35.61±0.06[e]	24.31±0.03[c]	1.89±0.01[b]	0.76±0.01[d]	0.31±0.01[b]	0.89±0.01[cd]
5	8.80±0.02[d]	4.48±0.01[b]	35.27±0.06[de]	24.34±0.03[c]	1.97±0.01[e]	0.75±0.01[bc]	0.32±0.01[e]	0.88±0.01[ab]
6	8.88±0.02[de]	4.60±0.01[cd]	36.56±0.09[f]	24.71±0.04[e]	1.94±0.01[cd]	0.75±0.01[cd]	0.32±0.01[cd]	0.88±0.01[ab]
7	8.61±0.02[b]	4.70±0.01[e]	35.62±0.06[e]	24.13±0.03[b]	1.84±0.01[a]	0.77±0.01[e]	0.29±0.01[a]	0.89±0.01[d]
8	8.96±0.02[e]	4.46±0.01[b]	35.52±0.06[e]	24.50±0.03[d]	2.01±0.01[f]	0.74±0.01[b]	0.34±0.01[f]	0.88±0.01[bc]
9	8.66±0.02[bc]	4.56±0.01[c]	35.16±0.07[d]	24.21±0.04[bc]	1.90±0.01[b]	0.76±0.01[cd]	0.31±0.01[b]	0.88±0.01[bc]
10	8.83±0.02[d]	4.59±0.01[c]	36.27±0.05[f]	24.67±0.03[e]	1.93±0.01[c]	0.75±0.01[bc]	0.32±0.01[c]	0.88±0.01[ab]
11	8.72±0.02[c]	4.47±0.01[b]	34.69±0.06[bc]	24.04±0.03[b]	1.96±0.01[de]	0.75±0.01[cd]	0.32±0.01[de]	0.88±0.01[bc]

SEM: standard error of the mean. Length [L, μm], Width [W, μm], Area [A, μm^2], Perimeter [P, μm], Ellipticity [L/W], Rugosity [$4\pi A/P^2$], Elongation [(L − W)/ (L + W)], Regularity [πLW/4A]. Total number of cells for each male: male 1 = 400, male 2 = 1000, male 3 = 600, male 4 = 800, male 5 = 800, male 6 = 400, male 7 = 800, male 8 = 800, male 9 = 600, male 10 = 1000, male 11 = 800. [a-f] Superscript indicates differences within column regarding boar. $P < 0.05$.

Table S2. Fertility variables (mean ± SEM) of litter size and mortality of piglets born after artificial insemination with semen of different boars.

	Total born per litter	Piglets born alive	Piglets born dead	Number of mummies	Litter weight at birth
1	9.80±0.09^{b}	8.77±0.09^{b}	0.86±0.03^{d}	0.17±0.02^{bcd}	14.12±0.13^{ab}
2	9.42±0.06^{a}	8.74±0.06^{b}	0.61±0.02^{c}	0.08±0.01^{a}	13.62±0.09^{a}
3	9.95±0.07^{b}	9.15±0.07^{c}	0.49±0.02^{b}	0.31±0.01^{f}	15.00±0.11^{c}
4	10.37±0.09^{c}	8.56±0.08^{b}	1.61±0.03^{f}	0.20±0.01^{cd}	14.85±0.13^{c}
5	9.96±0.06^{b}	9.34±0.06^{cd}	0.29±0.02^{a}	0.33±0.01^{f}	14.28±0.09^{b}
6	11.70±0.12^{e}	11.14±0.11^{f}	0.45±0.03^{b}	0.12±0.02^{ab}	18.81±0.18^{f}
7	11.76±0.06^{e}	10.33±0.06^{e}	1.02±0.02^{e}	0.41±0.01^{g}	16.83±0.09^{e}
8	9.25±0.06^{a}	8.50±0.06^{ab}	0.53±0.02^{bc}	0.22±0.01^{cde}	13.90±0.09^{ab}
9	10.37±0.07^{c}	9.42±0.06^{cd}	0.57±0.02^{bc}	0.39±0.01^{g}	15.77±0.09^{d}
10	10.79±0.05^{d}	9.57±0.05^{d}	0.96±0.01^{de}	0.26±0.01^{e}	16.10±0.09^{d}
11	10.01±0.06^{b}	8.84±0.06^{b}	1.01±0.02^{e}	0.16±0.01^{bc}	14.06±0.09^{ab}

SEM: standard error of the mean. Total number of cells for each male: male 1 = 400, male 2 = 1000, male 3 = 600, male 4 = 800, male 5 = 800, male 6 = 400, male 7 = 800, male 8 = 800, male 9 = 600, male 10 = 1000, male 11 = 800. Litter weight at birth (kg). $^{a-f}$ Superscript indicates differences within column regarding boar. $P < 0.05$.
